# The complete chloroplast genome sequences of an endangered orchid species *Paphiopedilum wenshanense* (Orchidaceae)

**DOI:** 10.1080/23802359.2022.2144514

**Published:** 2022-11-15

**Authors:** Hongying Liao, Jizheng Fan, Xiuling Li, Mingzhi Li, Yunfen Ning

**Affiliations:** a Flower Research Institute, Guangxi Academy of Agricultural Sciences, Nanning, China; b Bio&Data Biotechnologies Co. Ltd., Guangzhou, China; c College of Agriculture, Guangxi University, Nanning, China

**Keywords:** *Paphiopedilum wenshanense*, complete chloroplast genome, genome assembly, phylogenetic tree

## Abstract

*Paphiopedilum wenshanense*, a species of the family Orchidaceae, is endangered in the world with highly ornamental and biological value, the morphology of *P. wenshanense* is very similar to its relative species, *P. bellatulum* and *P. concolor*. However, there are few studies on the molecular biology and phylogeny of this species currently. Therefore, we report its complete chloroplast (cp) genome sequence at the first time, hoping to provide a foundation for its future phylogenetic analysis. The complete chloroplast genome of *P. wenshanense* was 161,750 bp in size, which contained a large single-copy (LSC) region of 90,656 bp, a small single-copy (SSC) region of 1886 bp, and two inverted repeat (IR) regions of 34,604 bp each. The total GC content was 35.66%. The genome encodes 38 transfer RNA genes, eight ribosomal RNA genes, and 80 protein-coding genes. The phylogenetic analysis indicated that the genetic relationship between *P. wenshanense* and *P. concolor* was very close.

*Paphiopedilum wenshanense* Z. J. Liu & J. Y. Zhang 2000 (family: Orchidaceae) is a lithophytic or terrestrial plant, which has a narrow distribution range, mainly distributed in the Nanpan River valley in Yunnan province, China (eFloras 2008; Chen et al. 2009). Due to the rare distribution in the wild, *P. wenshanense* was listed as class I protected plant in the List of National Key Protected Wild Plants in September 2021 (National Forestry and Grassland Administration & Ministry of Agriculture and Rural Affairs of the People’s Republic of China 2021). Furthermore, the morphology of *P. wenshanense* is very similar to that of the relative species, *P. bellatulum* and *P. concolor.* At present, the three related species can be roughly distinguished by the shape of the staminode. However, some individuals are still easily confused and very difficult to distinguish them accurately. To protect *P. wenshanense* better and provide significant genomic resources for the further study of Orchidaceae, we first reported its chloroplast (cp) genome and a maximum-likelihood (ML) phylogenetic tree was constructed for phylogenetic analysis.

The fresh green leaf of *P. wenshanense* was collected from Wenshan, Yunnan Province, China (N: 24°38′51″, E:104°36′95″). The plant materials and voucher specimens (the voucher number GXAAS-ORCHID-1784) were stored at the *Paphiopedilum Ex Situ* Conservation Germplasm Resource Nurseries of the Flower Research Institute, Guangxi Academy of Agricultural Sciences (http://www.gxaas.net/s.php/hhyjs/, Xiuling Li, E-mail: congzxiao@163.com). Genomic DNA was extracted using the DNeasy Plant Mini Kit (Qiagen, Valencia, CA). Following DNA extraction, 1 μg of purified DNA was fragmented and used to set up 250 bp short-insert libraries and the qualified libraries were sequenced with PE150 bp on a BGISEQ-500 sequencer according to the manufacturer’s instructions. Then DNA libraries were sequenced by Bio & Data Biotechnologies Inc. (Guangzhou, China) on the BGISEQ-500 platform with PE150 reads lengths. The cp genomes of *Paphiopedilum* from GenBank were used as seed sequence. We obtained a total of 5.1 G of raw data, and raw data were preprocessed by Fastp v.0.20.1 with default parameters (Chen et al. 2018) in order to trim adaptors and remove the low-quality RNA-seq reads. We used the program NOVOPlasty Version 3.8.3 (Dierckxsens et al. 2017) to assemble the clean reads. The cp genome was then annotated using the GeSeq (Tillich et al. 2017) and tRNAscan (Schattner et al. 2005), and was submitted to GenBank with the accession number MZ150830.

The complete cp genome of *P. wenshanense* was determined to comprise double-stranded, circular DNA of 161,750 bp containing two inverted repeat (IR) regions of 34,604 bp each, separated by a large single-copy (LSC) and a small single-copy (SSC) regions of 90,656 and 1886 bp, respectively. The genome contained 130 functional genes, including 80 protein-coding genes (PCGs), 38 tRNA genes, eight rRNA genes, and four pseudogenes. Eight PCGs, eight tRNA genes, four rRNA genes, and one pseudogene were fully duplicated in the IR region. Two copy genes (ccsA) were located on the IRb-SSC and IRa-SSC boundary, respectively. Eighteen genes contained two exons and four genes (clpP and ycf3 and two rps12) contained three exons. Two copies of the rps12 share the first exon in the LSC region and possess the second and third exons in the IR region, respectively. The overall GC content of *P. wenshanense* cp genome is 35.66%, with corresponding values of 32.91, 24.23, and 39.59% in the LSC, SSC, and IR regions, respectively.

The complete cp genome sequences of remaining 33 species were downloaded from GenBank and were selected to study the phylogenetic placement of *P. wenshanense*. The sequence alignment was conducted using MAFFT v7.475 (Katoh and Standley 2013), and then a ML phylogenetic tree (Figure 1) was constructed using FastTree version 2.1.10 with Generalized Time-Reversible (GTR) model, statistical support for branches was tested by Shimodaira–Hasegawa (SH) test (Price 2010). The results confirmed that *P. wenshanense* was closely related to *P. concolor*, with 98% support. In addition, the cp resources will still provide molecular genetic information for DNA future coding, conservation genetics, and *Paphiopedilum* breeding.

**Figure 1.**
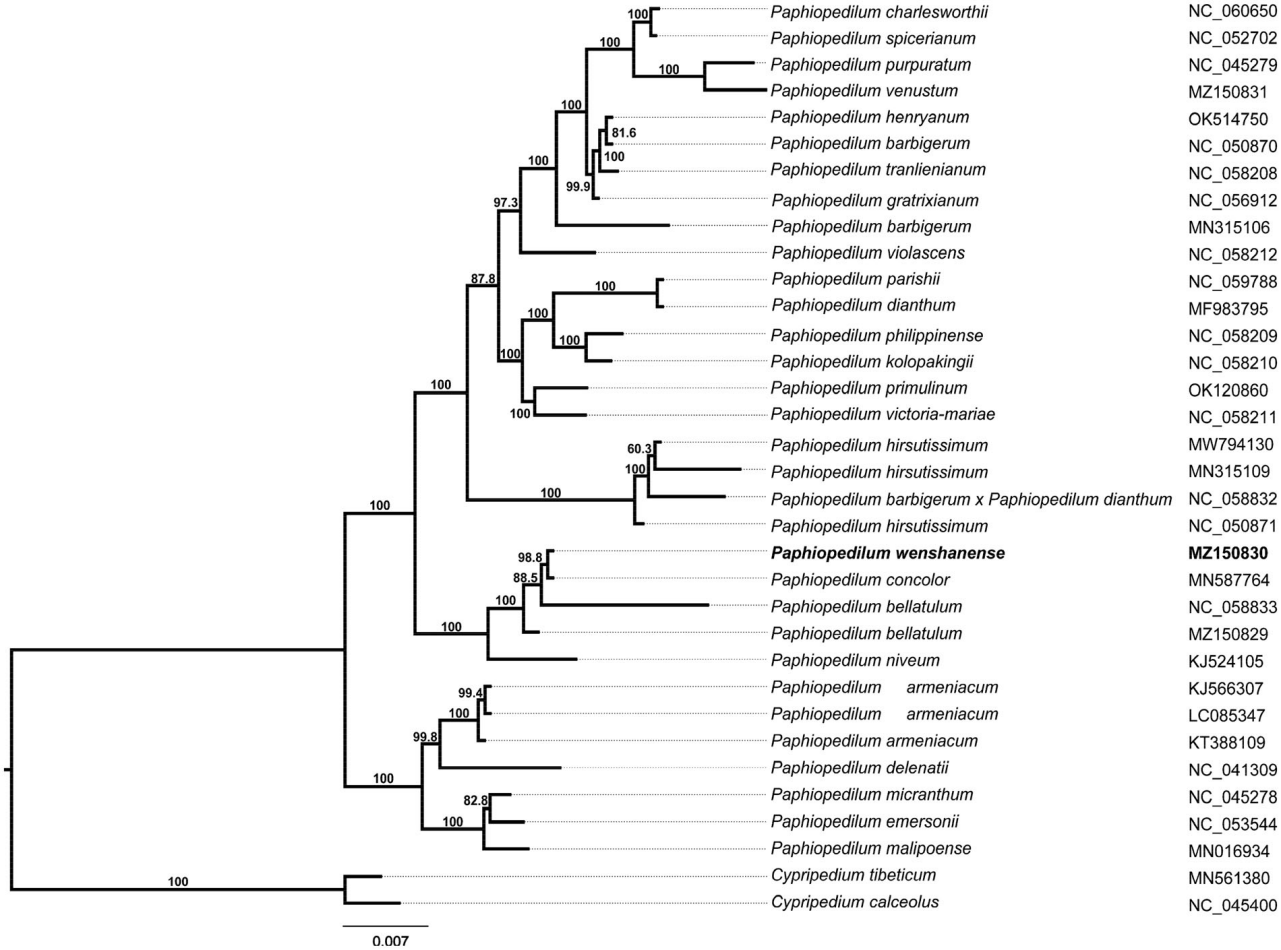
Maximum-likelihood phylogenetic tree based on complete chloroplast genomes from 32 *Paphiopedilum* plants and two *Cypripedium* plants and the support values are shown at the branches.

## Ethical approval

This article does not contain any studies with human participants or animals performed by any of the authors. We strictly comply with the Regulations of the People’s Republic of China on the Protection of Wild Plants, the International Union for Conservation of Nature (IUCN 2015) policies research involving species at risk of extinction, the Convention on Biological Diversity and the Convention on International Trade in Endangered Species of Wild Fauna and Flora.

## Data Availability

The genome sequence data that support the findings of this study are openly available in GenBank of NCBI at https://www.ncbi.nlm.nih.gov under the accession no. MZ150830. The associated BioProject, SRA, and Bio-Sample numbers are PRJNA746279, SRR15122878, and SAMN20199047, respectively.
